# Understanding Barriers and Facilitators to Disaster Preparedness in Federally Qualified Health Centers in the United States: A Mixed Methods Study

**DOI:** 10.1017/dmp.2024.102

**Published:** 2024-09-18

**Authors:** Saria Hassan, Michelle Wiciak, Karla Escobar, Myrna del Mar González Montalvo, Tess Richards, Hector Villanueva, Jean Ortiz, Dabney P. Evans, Marcella Nunez-Smith

**Affiliations:** 1 Department of Medicine, Emory University School of Medicine, Atlanta, GA, USA; 2 Hubert Department of Global Health, Emory Rollins School of Public Health, Atlanta, GA, USA; 3 St. Thomas East End Medical Center, St. Thomas, US Virgin Islands; 4 HealthProMed, San Juan, Puerto Rico, New Haven, CT, USA; 5 Department of Medicine, Yale School of Medicine, New Haven, CT, USA

**Keywords:** climate change, climate-related, extreme weather events, disasters, federally qualified health centers, health disparities, non-communicable disease

## Abstract

**Objective:**

Severe weather events exacerbate existing health disparities due to poorly managed non-communicable diseases (NCDs). Our objective is to understand the experiences of staff, providers, and administrators (employees) of Federally Qualified Health Centers (FQHCs) in Puerto Rico and the US Virgin Islands (USVI) in providing care to patients living with NCDs in the setting of recent climate-related extreme events.

**Methods:**

We used a convergent mixed-methods study design. A quantitative survey was distributed to employees at 2 FQHCs in Puerto Rico and the USVI, assessing experience with disasters, knowledge of disaster preparedness, the relevance of NCDs, and perceived gaps. Qualitative in-depth interviews explored their experience providing care for NCDs during recent disasters. Quantitative and qualitative data were merged using a narrative approach.

**Results:**

Through the integration of quantitative and qualitative data, we recognize: (1) significant gaps in confidence and preparedness of employees with a need for more training; (2) challenges faced by persons with multiple NCDs, especially cardiovascular and mental health disorders; and (3) most clinicians do not discuss disaster preparedness with patients but recognize their important role in community resilience.

**Conclusion:**

With these results, we recommend strengthening the capacity of FQHCs to address the needs of their patients with NCDs in disasters.

At least 30% of mortality after climate-related extreme weather events (disasters) have been attributed to poorly managed non-communicable diseases (NCDs).^1^ The excess mortality seen after Hurricanes Irma and Maria hit Puerto Rico and the US Virgin Islands (USVI) is a testament to the importance of addressing the needs of people living with NCDs in disaster settings.^2^ Unfortunately, with the advent of climate change, the world will experience an increase in the frequency of severe climate-related extreme weather events.^3^ The Caribbean is a region with high exposure to these events.^4,5^

Vulnerability to climate change and climate-related extreme weather events can be considered a function of exposure, sensitivity, and adaptive capacity.^6^ In addition to the higher level of exposure described previously, the Caribbean region, with the highest level of premature mortality due to NCDs in the Americas, has a heightened level of sensitivity to the impact of climate-related events.^7^ As we consider how to strengthen adaptive capacity (ability to withstand the impact of climate change on health), we must better understand the challenges faced by the providers of health care services to persons living with NCDs.

We engaged government-funded federally qualified health centers (FQHCs) in the USVI and Puerto Rico that provide care for the uninsured, underinsured, and most vulnerable populations. Our objective was to understand the experiences of staff, providers, and administrators of FQHCs in Puerto Rico and the USVI in providing care and services to patients living with NCDs in the setting of recent climate-related events.

## Methods

### Study Design and Setting

A concurrent convergent mixed-methods study design was used to understand the experience of persons working in FQHCs in the US territories in addressing the needs of their patients and staff living with NCDs in the setting of a disaster and how to improve preparedness moving forward. Study participants were recruited from 2 FQHCs in the US territories of Puerto Rico and the USVI severely affected by the 2017 Hurricanes Irma and Maria.

### Inclusion Criteria

The inclusion criteria for the quantitative and qualitative study included currently employed at the selected FQHCs, present on island and experienced the 2017 hurricanes (did not have to be working for the FQHC at the time), and able to provide informed consent.

### Participant Recruitment

Study purpose and scope were presented to employees at the “staff and provider meeting” at both FQHCs. A QR code to complete the survey was provided at the end of the presentation. The survey was also sent to the provider/staff listserv. At the end of the quantitative survey, participants were asked if they would be interested in a qualitative interview. Those who indicated “yes” and provided contact information were contacted for an in-depth interview.

### Quantitative Methods

Survey data were collected and managed using REDCap electronic data capture tools hosted at Emory University.^8,9^ The survey domains assessed included age, gender, role, length in role, working at the FQHC during the hurricanes in 2017, role during emergency response, emergency preparedness at their FQHC, level of preparedness, knowledge and confidence regarding natural disaster response, and the needs of vulnerable patients. Our community and scientific advisory group reviewed and piloted the survey.

Dependent Variables: Our Disaster Plan Knowledge Composite (DPKC) scores were created by summing 4 questions regarding confidence in dealing with natural disasters: (1) Do you have knowledge of the evacuation plan at your center? (2) Do you have knowledge of the Shelter in Place Plan for your center? (3) Do you know when to use “Drop, Cover, and Hold On?” (4) Do you know where to find information about your center’s emergency response plan? The first 2 and last questions were yes/no responses, where “yes” was coded as “1” and “no” was coded as “0.” The “Drop, Cover, and Hold On” question was scored “1” for the correct answer and “0” for other answers, including “I don’t know.” DPKC scores ranged from 0 to 4, with “0” being the lowest composite knowledge score and “4” being the highest. Percentiles were used to help categorize DPKC scores, and the scores were sorted into low (DPKC scores 0–2), intermediate (DPKC score 3), and high (DPKC score 4).

Levels of preparedness and confidence were dichotomized using responses to the question: “How prepared do you feel for a hurricane?” Low preparedness included “mostly unprepared,” “a little unprepared,” “neutral,” or “a little prepared,” and high preparedness included “mostly prepared” or “completely prepared.” Responses to the question: “How confident do you feel that you know what to do in the event of a hurricane at work, outside of work” included “not confident at all,” “a little not confident,” “neutral,” “a little confident,” and high confidence included “mostly confident” and “very confident.”

Independent Variables: age, gender, role, length in role, and whether they worked at the FQHC during the hurricanes in 2017. All participants were also asked the question: “do you talk to patients regularly about how to prepare for a disaster” with responses as “yes,” “no,” and “I don’t interact with patients.” This question was analyzed separately because 26.4% reported they did not interact with patients which made it not relevant to them.

Analysis was done using SPSS version 27.0 (IBM Corp, Armonk, NY, USA). All statistics were conducted at the 95% confidence level with a significance level set at alpha = 0.05. A data analysis included descriptive statistics, Fisher’s exact tests, and chi-square analyses. We removed missing data from the overall data analysis.

### Qualitative Methods

Participants in qualitative, semi-structured, in-depth interviews were recruited through purposive sampling methods in collaboration with participating FQHCs. Purposive sampling was used in addition to those identified through the survey (see above) to ensure a diversity of experiences in selecting for a range of staff jobs (physicians, administrators, others), gender, and age.

One-hour interviews were conducted with participants by trained research assistants with experience in qualitative interviewing methods. All interviews were conducted virtually via Zoom given the coronavirus disease (COVID-19) pandemic and safety concerns in Puerto Rico and USVI. Interviews for participants in Puerto Rico were conducted in Spanish. Interviews were recorded and transcribed. Transcripts were de-identified and securely stored.

The analysis of transcripts was done by SH, KE, and MG, using thematic content analysis. Each transcript was analyzed by 2 persons with a third review as needed. Iterative inductive coding was used until thematic saturation was reached.^10^ Dedoose software was used to facilitate the analysis and finalization of a codebook. Emergent themes were discussed and agreed upon.

#### Data Integration

Once quantitative and qualitative data were analyzed separately, we integrated results to expand our understanding of the management of NCDs in disasters. To do this, we compared the 2 data sets. We looked for common concepts across the data sets and then displayed the common concepts, with the corresponding quantitative and qualitative data, using a joint display. We then interpret the side-by-side comparison of the quantitative and qualitative data, using a narrative approach. This allows us to compare the results of merging data sets, using a joint display format, and then use a narrative approach that brings out discordance, concordance, and expansion of our understanding of the data.^11,12^

#### Ethical Consideration

The Emory University Institutional Review Board reviewed and approved this study (study# 00001518). All study participants consented in English or Spanish to both the survey and in-depth interviews.

## Results

### Quantitative Results

Out of 164 responses, 14 participants were removed as they answered eligibility questions but did not start the survey. Thus, in total, 150 participants were included in a statistical analysis based upon inclusion/exclusion criteria with a 76% response rate. A summary of demographic information is provided in Table 1.

Over 56% of respondents were either administrators, nurses, or physicians at the FQHC, and nearly 29% of respondents have been in their current role for over 5 years (see Table 1). Fifty percent (n = 75) of respondents were employees during Hurricanes Irma and Maria in 2017 (see Table 1). Only 34.4% of participants (n = 33) were a part of the emergency response team with 40% of those (n = 10) in the command/leadership role and 28.0% of those (n = 7) in the operations role. The top 3 NCDs deemed most vulnerable during a natural disaster were heart failure (n = 90), mental health disorders (n = 89), and diabetes (n = 77). Seventy-five percent of participants who interact with patients reported that they do not speak to patients about disaster preparedness (n = 69 out of 92).

Nearly 73% of participants (n = 59) had low preparedness for the hurricanes in 2017 (see Table 1). It was found that preparedness for the 2017 hurricanes was associated with age (*P* = 0.007) and role during the emergency response (*P* = 0.015) (Table 2).

Slightly over half of the participants (n = 76) had low confidence of dealing with a natural disaster if stuck at work (see Table 1), and it was found that the confidence of dealing with a natural disaster at work was associated with FQHC site location (*P* < 0.001), role during the emergency response (*P* = 0.025), and last time the participant attended an emergency preparedness training (*P* = 0.037) (see Table 2).

Half (50.4%) of the participants (n = 68) had low confidence of dealing with a natural disaster outside of work (see Table 1), and it was found that the confidence of dealing with a natural disaster outside of work was associated with the FQHC location site (*P* < 0.001) and its role during the emergency response (*P* = 0.010) (see Table 2).

The average DKPC score was 2.83 (SD = 0.93, n = 96) with sub-composite frequencies in Table 1. Overall, only 28% of the participants (n = 27) met criteria for high DPKC scores. There was no statistically significant assocation between DPKC scores and any of the independent variables (see Table 2).

When asked what leaders could do to better prepare staff and providers, 56.7% (n = 85) wanted practice exercises, 43.3% (n = 65) wanted in-person training, 36.7% (n = 55) wanted information for patients, and 26.7% (n = 40) wanted virtual training.

### Qualitative Results

The research team conducted 17 semi-structured, in-depth interviews across FQHCs that included 10 females and 7 males. Their roles consisted of 5 administrative staff, 8 providers, and 4 participants with both provider and administrative roles. After the analysis, 4 major themes emerged (Table 3): the difficulty of managing NCDs in the setting of a disaster, high mental health burden for community and staff following a disaster, gaps in provider training and emergency planning, and the power of community resilience and response.

#### Theme 1: Staff experienced difficulties managing NCDs in a disaster

Interviewees recounted challenges experienced by patients with diabetes, hypertension, asthma, heart conditions, and mental health problems. Emerging subthemes included *barriers to accessing medication, treatment, and fresh foods for NCD management* and *staff viewed NCD self-management as important but not prioritized by patients.*

##### Barriers to accessing medications, treatment, and fresh foods for NCD management.

Disasters impacted the management of NCDs through disrupted access to services and other resources. Barriers were a direct result of physical barriers (eg, destruction of roads and closing of buildings), the limited availability of power alternatives for medical equipment or to keep medications at appropriate temperatures, and the limited availability of health services, medications, and fresh foods. The destruction of roads and buildings created transportation challenges for patients seeking services and for outreach groups to reach patients in need, especially non-ambulatory patients.

The limited medication access was a problem for many patients living with NCDs:

There were many patients that were, that were left without their medications for a long time including chronic patients with diabetes, cardiac conditions, hypertension and so we had to provide their medications, control their conditions so that their health would improve. (HPM 7)

Most interviewees discussed the limited access to fresh foods, especially patients with diabetes. A few interviewees described how a nutritious, balanced diet is central to the management of diabetes, and lack of fresh foods negatively impacted the health of patients with diabetes:

And these diabetic patients that needed treatment as well as consistent meals, all these things and when they didn’t have these provisions it was very difficult, uh, to manage. And I would tell you that there were no resources because there was no food. (HPM 5)

Another participant described a similar challenge for patients with diabetes:

...‘Cause like I said, people did not have access to their medications. Um, they didn’t have access to good foods or healthy foods. Um, even those patients who typically don’t eat all that great to begin with, it was worsened by this because what they would get in those pre-formed things was just, you know, all this processed, sodium-packed food, M&Ms and Butterfingers and Twix bars in their little bags. (USVI 5)

##### Staff viewed NCD self-management as important but not prioritized by patients.

Many interviewees explained that following the destruction caused by a disaster, people were in *survival mode.* Depending on individuals’ circumstances and what they endured during the disaster (eg, destruction of home, loss or injury to loved ones), patients with NCDs and the community were sometimes more concerned with finding water, food, shelter, and other necessary items for survival. Medications or other supplies necessary for the management of their NCDs were not considered essential:

And then, you know, it’s hard enough having them to be compliant on a regular basis, but when people are going through, their whole mindset and their perception changes because, now, they’re in survival mode. And survival mode does not always entail, for them, taking my medication. Survival mode is havin’ water, bein’ able to take a bath, be able to put on so clean clothes, bein’ able to put some food in my mouth. But they’re not necessarily thinkin’ on the health. (USVI 3)

Interviewees explained that patients are responsible for their well-being. During and after a disaster, this responsibility entails ensuring adequate medications and supplies needed to manage their conditions and knowing where to seek health care if needed:

....on my end as a patient, I would prepare my equipment in case of an emergency. Because there are times, that come what may, come a hurricane, a storm, and [patients], I mean, they don’t prepare. So we give our all but they also need to help because here they are the patients. (HPM 6)

#### Theme 2: High mental health burden for community and staff following a disaster

Most interviewees mentioned the high mental health burden related to traumatic experiences and their shared responsibilities between home and work. The subthemes included: *staff witnessed a high burden of mental health needs after a disaster* and *staff felt a double burden of caring for patients as well as themselves and their families*.

##### Staff witnessed a high burden of mental health needs after a disaster.

Most interviewees explained that there was a high need for mental health services after the disasters due to trauma experienced by both the community and health staff:

There were many cases, uh, of anxiety, depression, uh a lot of anguish, that well, right, they relieved many of the situations and fears and anguish that they lived through during Maria, from hurricane Maria. (HPM 2)

Interviewees shared stories of themselves, family, or patients who experienced losing everything, including their home and the death of loved ones, lack of means of communication, lack of electricity, and the scarcity of water and food needed to survive. These circumstances resulted in an acute trauma requiring mental health services; support was needed for community members to deal with feelings of anxiety, depression, anguish, fear, desperation, and anger. For example, 1 interviewee shared a story of a co-worker who was traumatized by the fear of almost losing her daughter during the storm:

I remember this one staff member, um, the-the door of her house blew off and her daughter was getting pulled, and she had to pull her daughter back in. I mean so she was I mean completely traumatized by this. Um, and then when, you know, 12 days later, now Maria hits, she was horrified. She was terrified that something else was going to happen again. (USVI 5)

For the community, interviewees identified stressors that highlighted the need for readily available mental health services for patients. Many patients who were simultaneously caretakers of loved ones (eg, older adult parents) experienced greater stress and anxiety. This was due to having to move their loved ones during the disaster or concerns about not being unable to care for them properly:

There were many cases, uh, of anxiety, depression, uh a lot of anguish, that well, right, they relieved many of the situations and fears and anguish that they lived through during Maria, from hurricane Maria. (HPM 2)

##### Staff felt a double burden of caring for patients as well as themselves and their families.

Most interviewees raised the issue of balancing responsibilities for their family, themselves, and work, as providers for the community when disasters hit:

So, of course, you know, when-when it’s a time like that, you always think of family first. But then, you know, we’re in the business of service—and so you can’t put—you can’t lab—uh, label family and service. You have to put them on the same playin’ field—when you take that responsibility. (USVI 3)

A few participants also discussed the importance of taking care of themselves before they can focus on providing good care to patients. Self-care included resting, having proper nutrition, and minimizing stressors at home:

...means that you are eating well; you got some rest; and, um, at home is as stable as possible. Because to come to work and function and knowing at home is not okay, it defeats the-the-the purpose of you being there and giving as much as you can give. Um once you are in that position, then you can think better; you could respond better, your attitude is better because things change in a disaster. Um, so I-I think, um, taking care of yourself emotionally and physically before you’re coming. (USVI 1)

#### Theme 3: Staff identified gaps in provider training and emergency planning

Interviewees discussed the importance of effective provider education on disaster preparedness and response. Written plans are important in preparing and helping direct staff during the disaster response:

Um, it’s one thing to have the plan written... but many people are not going to run for the book, and the-the you know, the disaster plan. The plan, written plan is important, so are the contacts executing what’s in the plan is very important because without a plan, you know, things, you know, things might be missed. (USVI 1)

Practical drills and training were also critical to learning. Providers and health staff were offered training on various natural disaster topics, such as hurricanes and earthquakes and other natural disasters. Many of the offered trainings were online video learning modules.

Training was supplemented with disaster preparedness material via informative emails at the start of the hurricane season or as a storm approached.

Participants acknowledged that provider training and the FQHC emergency plan could be improved. There was a lack of tabletop (practical) exercises in current disaster training, and a few participants recognized the need to expand their written emergency plan with greater detail:

...when a storm hits, this is what happens. This is who we call. This is where we meet. If that place is destroyed, this is the second places that-that we meet, and this is how we go through things. [I tell admin] we need to start getting these-these things written down and getting the staff trained about it, but we still don’t have anything that’s that detailed. (USVI 5)

Another limitation was that the FQHC emergency plans did not encompass the needs of patients with NCDs or other vulnerable populations (eg, homeless, non-ambulatory, older adult population):

And th-that’s the part that I think is really missing. Like I said, we can figure out maybe how to get back into the facility, but I don’t think we have, um, enough preparation for taking care of our patients with chr-with-with chronic disease. (USVI 5)

While there were a few participants who shared a preference for virtual training due to its convenience, many preferred in-person. Interviewees were in general agreement that higher frequency and in-person training would improve disaster preparedness training since they were more interactive and of higher quality. One participant also stressed the need to listen to the community’s feedback to improve their emergency response.

#### Theme 4: Staff emphasized the power of community resilience and response

All participants discussed the commitment that the FQHCs staff had for patients and communities after the disaster:

But, um, I must say that, in the Caribbean, we are pretty much used to that, um—we’re pretty much service oriented. And because the communities are so close-knit, it’s not just, um, about service for us. It’s also that we feel like we’re serving our own, for some reason. And it-it-it’s more impacting. And so we have that drive that, by any means necessary, whatever has to be done has to be done. (USVI 3)

There were stories of the community coming together to share their resources and help one another after the disaster. Providers committed to serving their patients, despite the many challenges they faced accessing them:

Then every day too we weren’t able to get the cars out or anything clearly because all of the roads were covered with debris and telephone lines and trees and logs and everything else, boulders, and water was gushing everywhere for 40 days, plus 40 days, and every day I was walking in sneakers ’cause those were pretty much the only shoes I had ’cause everything else blew away. They were soft and wet because I walked through the mud and everything to the hospital to make sure I was there... (USVI 4)

Interviewees discussed how disaster response by the FQHC was flexible and adapted to the needs of the population along with the central role of the FQHC in providing services and support to their community. This included assessing the damage of the storm, strategizing ways to overcome those challenges, and using outreach to provide care to patients in need. One health center also took on the role of coordinating help with external organizations to provide services (eg, food) directly to the communities.

### Mixed Methods

Data integration of qualitative and quantitative information was done using merging with results displayed side by side in Table 4. We interpret the comparison of quantitative and qualitative data using a narrative approach where the key outcomes of the integration are the following:

1. There is a need for improved emergency preparedness planning and training in the FQHCs to improve staff and provider confidence and knowledge. This training and preparedness planning will need to include practical in-person exercises and consider the double burden suffered by providers in the setting of disasters.
2. Employees agree that the persons with NCDs are among the most vulnerable in the setting of a disaster. Given challenges faced by persons living with NCDs in disasters, it is important for their patients to have the ability to self-manage their condition and to access acute mental health services and support.
3. Despite the lack of active engagement of their patients in discussions around disaster preparedness, health center staff and providers recognize the important role they play in educating patients on the importance of preparedness, especially those with chronic disease. This is directly related to their contributions to the resilience of the community.

## Discussion

We have described the experience of health centers serving the most vulnerable in the aftermath of Hurricanes Irma and Maria in Puerto Rico and the USVI. Our use of a mixed-methods approach has provided us with an opportunity to quantify levels of preparedness and areas for capacity strengthening, and to qualify how that relates to challenges and opportunities in preparing for climate-related events at the institutional and individual levels.

Our work corroborates findings from other papers that have identified the significant challenge of managing NCDs in the immediate aftermath of a disaster. Andrade *et al.*, in their qualitative study with diverse stakeholders, identified similar challenges including access to care, medication, and mental health concerns.^13^ In their scoping review focusing on the impact of climate change on persons living with diabetes, Ospelt *et al*. identified the role of climate-related extreme weather events on insulin storage, access to diabetes supplies, and maintaining a special diet.^14^ Mellgard *et al.*, in their 2019 paper, discussed the policy implications of challenges managing NCDs in disasters specifically as they relate to NCDs^15^; this includes ensuring access to care for persons with complex medical conditions. Our paper, however, fills a gap in the literature that seeks to address the view of providers in health centers in providing preparedness and response services in the setting of climate-related extreme events.

The findings from our robust mixed methods assessment support the following recommendations to strengthen the adaptive capacity of health centers to address the needs of their patients living with NCDs in the setting of a disaster:

1. Continued regular disaster preparedness training that incorporates more in-person learning and drills. The role of in-person and practice-based skill training for disaster preparedness is critical. Emergency preparedness planning needs to also take into account the double burden on providers/staff and ensure the equitable rotation of clinic shifts to allow time for providers to care for their loved ones and homes.
2. Providing resources to clinicians to facilitate discussions around disaster preparedness for patients living with NCDs. The health centers and providers can play a critical role in educating patients on the special preparedness needs they have given their chronic health condition. Facilitating these discussions and communicating the importance of disaster preparedness for these populations.
3. Availability of mental health support services for staff, providers, and their patients. The mental health needs of all persons experiencing a disaster are significant. One cannot start to address other chronic care needs if the immediate trauma of the event is not managed. Having staff and community members trained to provide assistance through tools such as Psychological First Aid can help address this critical need immediately after a disaster.
4. Engaging communities and community leaders to develop systems to support community members with NCDs and other vulnerabilities. Health centers should leverage the power of community and social support to address the needs of persons living with NCDs in disasters. Through partnership and identification of individuals in communities with additional needs due to chronic illnesses or immobility, the community can ensure support for those persons when disasters hit.

### Limitations

Despite the strengths of this mixed methods study in providing concrete recommendations for strengthening adaptive capacity for persons living with NCDs, there are some limitations to note. First, the study was conducted in US territories, so the generalizability of findings must be carefully considered. The US territories of Puerto Rico and the USVI hold similarities and differences to the mainland United States and to other Caribbean islands. In addition, our survey response rate of 60%, while adequate, does mean that we did not capture the views of all staff and providers. Similarly, interviews, while reaching thematic saturation, may not have captured all views.

## Conclusion

In conclusion, FQHCs play a vital role in the ability of vulnerable persons living with NCDs to adapt to the rising incidence and severity of climate-related extreme weather events. Despite experiences with climate-related extreme events, there continues to be a need for capacity strengthening to build the confidence and skills of frontline workers in managing NCDs during disasters.

## Figures and Tables

**Table 1.** Demographic summaries

	n	%
**Location**		
USVI	68	45.3
PR	82	54.7
**Age range**		
20–39	45	37.5
40–59	57	47.5
60+	18	15.0
**Gender**		
Man	23	19.3
Woman	92	77.3
Non-binary	3	2.5
Prefer to self-describe	1	0.8
**Current role**		
Administrator	33	24.8
Nurse	25	18.8
Physician and health care provider	23	17.2
Front desk staff	12	9.0
Other (including radiographer and nutritionist)	10	7.5
Dental provider	8	6.0
Patient assistant	8	6.0
Pharmacy professional	8	6.0
Social worker, mental health provider	6	4.5
**Length of time in current role**		
0–2 years	61	40.7
3–5 years	43	28.7
6–8 years	13	8.7
9–10 years	10	6.7
> 10 years	23	13.3
**Worked during the hurricane**		
Yes	75	50.0
No	75	50.0
**Worked during the earthquake (PR only)**		
Yes	61	74.4
No	21	25.6
**Has your supervisor or anyone else in the organization spoken to you about emergency preparedness?**		
Yes	122	88.4
No	16	11.6
**When was the last time you attended a training on emergency response/ preparedness at your organization?**		
0–6 months	66	49.6
7–12 months	22	16.5
13–18 months	8	6.0
19–24 months	4	3.0
24+ months	2	1.5
Never attended a training	10	7.5
Can’t remember	21	15.8
**Preparedness for 2017 natural disaster**		
Mostly unprepared	6	7.4
A little unprepared	6	7.4
Neutral	19	23.5
A little prepared	28	34.6
Mostly prepared	16	19.8
Completely prepared	6	7.4
**Confidence in dealing with a natural disaster while at work**		
Not confident at all	5	3.7
A little not confident	13	9.6
Neutral	16	11.9
A little confident	42	31.1
Mostly confident	42	31.1
Very confident	17	12.6
**Confidence in dealing with a natural disaster outside of work**		
Not confident at all	5	3.7
A little not confident	9	6.7
Neutral	17	12.6
A little confident	37	27.4
Mostly confident	43	31.9
Very confident	24	17.8
**Patients most vulnerable during a natural disaster**		
Heart failure	90	72.6
Mental health disorders	89	71.8
Diabetes	77	62.1
Functional disability	71	57.3
Asthma	70	56.5
Hypertension	62	50.0
Other	5	4.0
**Disaster Plan Knowledge Composite (DPKC) scores**		
Low (scores 0–2)	36	37.5
Intermediate (score of 3)	33	34.4
High (score of 4)	27	28.1

**Table 2.** Associations found for preparedness and confidence for disasters

Preparedness for the most recent disaster				Confidence in knowing what to do during a disaster while at work			Confidence in knowing what to do during a disaster while at home			DPKC composite scores			
	Low preparedness % (n)	High preparedness % (n)	*P*	Low confidence % (n)	High confidence % (n)	*P*	Low confidence % (n)	High confidence % (n)	*P*	Low % (n)	Intermediate % (n)	High % (n)	*P*
**Site**													
USVI	65.7 (23)	34.3 (12)	0.209	40.6 (26)	59.4 (38)	<0.001	32.8 (21)	67.2 (43)	< 0.001***	35.3 (12)	32.4 (11)	32.4 (11)	0.792
PR	78.3 (36)	21.7 (10)		70.4 (50)	29.6 (21)		66.2 (47)	33.8 (24)		38.7 (24)	35.5 (22)	25.8 (16)	
**Sex**													
Male	66.7 (10)	33.3 (5)	0.443	40.9 (9)	59.1 (13)	0.322	36.4 (8)	63.6 (14)	0.392	35.0 (7)	30.0 (6)	35.0 (7)	0.620
Female	76.4 (42)	23.6 (13)		57.1 (52)	42.9 (39)		51.6 (47)	48.4 (44)		36.8 (25)	35.3 (24)	27.9 (19)	
Non-binary	50.0 (1)	50.0 (1)		33.3 (1)	66.7 (2)		33.3 (1)	66.7 (2)		0.0 (0)	100.0 (2)	0.0 (0)	
Other	-	-		-	-		-	-		-	-	-	
**Age range**													
20–39	92.9 (26)	7.1 (2)	0.007**	57.8 (26)	42.2 (19)	0.197	53.3 (24)	46.7 (21)	0.419	43.2 (16)	37.8 (14)	18.9 (7)	0.438
40–59	56.7 (17)	43.3 (13)		45.6 (26)	554.4 (31)		42.1 (24)	57.9 (33)		30.0 (12)	35.0 (14)	35.0 (14)	
60+	73.3 (11)	26.7 (4)		68.8 (11)	31.3 (5)		56.3 (9)	43.8 (7)		26.7 (4)	33.3 (5)	40.0 (6)	
**Role**													
Physician/health care provider	78.6 (11)	21.4 (3)	0.817	52.2 (12)	47.8 (11)	0.300	52.2 (12)	47.8 (11)	0.821	47.4 (9)	31.6 (6)	21.1 (4)	0.185
Nurse	71.4 (10)	28.6 (4)		52.2 (12)	47.8 (11)		43.5 (10)	56.5 (13)		37.5 (6)	12.5 (2)	50.0 (8)	
Administrator	75.0 (15)	25.0 (5)		48.4 (15)	51.6 (16)		48.4 (15)	51.6 (16)		30.0 (6)	35.0 (7)	35.0 (7)	
Front desk staff	83.3 (5)	16.7 (1)		83.3 (10)	16.7 (2)		66.7 (8)	33.3 (4)		25.0 (2)	75.0 (6)	0.0 (0)	
Other providers	88.9 (8)	11.1 (1)		56.3 (9)	43.8 (7)		50.0 (8)	50.0 (8)		40.0 (4)	40.0 (4)	20.0 (2)	
Other support staff^	60.0 (6)	40.0 (4)		70.6 (12)	29.4 (5)		58.8 (10)	41.2 (7)		38.5 (5)	46.2 (6)	15.4 (2)	
**Working at the FQHC during the hurricanes in 2017**													
Yes	72.1 (44)	27.9 (17)	0.802	61.4 (43)	38.6 (27)	0.212	50.0 (35)	50.0 (35)	0.929	26.7 (12)	35.6 (16)	37.8 (17)	0.064
No	75.0 (15)	25.0 (5)		50.8 (33)	49.2 (32)		50.8 (33)	49.2 (32)		47.1 (24)	33.3 (17)	19.6 (10)	
**Part of emergency response team**													
Yes	67.7 (21)	32.3 (10)	0.331	50.0 (15)	50.0 (15)	0.113	40.0 (12)	60.0 (18)	0.088	19.0 (4)	33.3 (7)	47.6 (10)	0.091
No	77.6 (38)	22.4 (11)		67.2 (41)	32.8 (20)		59.0 (36)	41.0 (25)		37.8 (17)	40.0 (18)	22.2 (10)	
**Role during emergency response**													
Command/leadership	70.0 (7)	30.0 (3)	0.015*	60.0 (6)	40.0 (4)	0.025*	30.0 (3)	70.0 (7)	0.010**	14.3 (1)	57.1 (4)	28.6 (2)	0.481
Operations	71.4 (5)	28.6 (2)		71.4 (5)	28.6 (2)		85.7 (6)	14.3 (1)		28.6 (2)	14.3 (1)	57.1 (4)	
Logistics	100.0 (3)	0.0 (0)		100.0 (3)	0.0 (0)		66.7 (2)	33.3 (1)		0.0 (0)	0.0 (0)	100.0 (2)	
Planning	0.0 (0)	100.0 (5)		0.0 (0)	100.0 (5)		0.0 (0)	100.0 (5)		0.0 (0)	0.0 (0)	100.0 (1)	
**Last time attended emergency preparedness training**													
Never attended	0.0 (0)	0.0 (0)	0.548	70.0 (7)	30.0 (3)	0.037*	50.0 (5)	50.0 (5)	0.252	50.0 (3)	50.0 (3)	0.0 (0)	0.105
Within the past year	73.6 (39)	26.4 (14)		46.6 (41)	53.4 (47)		43.2 (38)	56.8 (50)		29.2 (19)	35.4 (23)	35.4 (23)	
More than a year ago	80.0 (8)	20.0 (2)		83.3 (10)	16.7 (2)		66.7 (8)	33.3 (4)		45.5 (5)	27.3 (3)	27.3 (3)	
Cannot remember	60.0 (9)	40.0 (6)		66.7 (14)	33.3 (7)		61.9 (13)	38.1 (8)		64.3 (9)	28.6 (4)	7.1 (1)	

* Significant at *P* < 0.05

** Significant at *P* < 0.01

*** Significant at *P* < 0.001

^ Includes IT, opticians, director of clinical laboratory, information management, radiographers, and a nutritionist

- Suppressed data, n < 5

**Table 3.** Emergent themes from in-depth interviews

Main theme	Subthemes	Description
Staff experienced difficulties managing NCDs in a disaster.	– Barriers to accessing medication, treatment, and fresh foods for NCD management – NCD self-management as important but not prioritized by patients	Challenges arising during and immediately after a disaster impact access to care, medication, and equipment that exacerbate symptoms for NCD patients. In response, FQHCs adapted and provided outreach to bring critical services to the community.
High mental health burden for community and staff following a disaster.	– Significant mental health needs after a disaster – Double burden on staff of caring for patients and their own families after a disaster	Mental health services are critical to both the community and FQHC staff since both staff and patients experience trauma during and after a disaster. After a disaster, FQHCs prioritize the well-being of their staff to ensure that they can effectively provide services to their patients.
Gaps in provider training and emergency planning.	– Need for practical exercises and drills – Need to incorporate NCD needs	There are many gaps in current FQHC emergency plans, and there is a need for greater disaster training. Higher frequency and in-person training can improve provider disaster preparedness education.
The power of community resilience and response.	– The role of the FQHC as a source of support – The role of the community as a source of support	The commitment of FQHC providers and staff to the betterment of their communities and bringing health care access to the most vulnerable. Neighbors aiding and offering support to others in the community immediately following a disaster.

**Table 4.** Narrative integration of quantitative and qualitative data

Summarized quantitative results	Corresponding themes from qualitative analysis	Integration of quantitative and qualitative data using a narrative approach
*Nearly half of respondents continue to have a low level of confidence in their preparedness levels and confidence in managing in the setting of natural disasters.*	The need to address gaps in the FQHC emergency plan The important role of provider education	There are clear gaps in preparedness levels and knowledge of emergency planning. There is a continued need for emergency preparedness planning and training in the FQHC so that staff and provider confidence and knowledge improve. This training and preparedness planning will need to take into account the double burden suffered by providers in the setting of disasters.
*Seventy-three percent believe heart failure patients are the most vulnerable, followed by 72% believing that mental health patients are the most vulnerable.*	The importance of access to NCD management The important role of mental health	Staff and providers agree that the most vulnerable in the setting of a disaster are those with chronic NCDs. There is an acknowledgment of the importance of accessing NCD management in disasters and the important role of mental health in these settings.
*Seventy-five percent of providers do not talk to patients about NCDs in disasters.*	The double burden on providers: caring for self, family, and patients The power of community resilience and response	Despite the lack of active engagement of patients in discussions around disaster preparedness, health center staff and providers recognize the important role they play in educating patients on the importance of preparedness, especially those with a chronic disease. This is directly related to their contributions to the resilience of the community. Providers, in particular, recognize this role but at the same time acknowledge the increased burden it puts on them.

## Abbreviations

CMS: Centers for Medicare and Medicaid
EP: emergency preparedness
FQHC: federally qualified health center
NCD: non-communicable disease
USVI: US Virgin Islands
